# Identification of the Potential Metabolic Pathways Involved in the Hepatic Tumorigenesis of Rat Diethylnitrosamine-Induced Hepatocellular Carcinoma via ^1^H NMR-Based Metabolomic Analysis

**DOI:** 10.1155/2019/9367082

**Published:** 2019-01-02

**Authors:** Minjiang Chen, Siming Lu, Hong Zheng, Min Xu, Jingjing Song, Weibin Yang, Qiaoyou Weng, Liyun Zheng, Xiaoxi Fan, Xingyao Cheng, Hongchang Gao, Jiansong Ji

**Affiliations:** ^1^Key Laboratory of Imaging Diagnosis and Minimally Invasive Intervention Research, the Fifth Affiliated Hospital of Wenzhou Medical University/Affiliated Lishui Hospital of Zhejiang University/The Central Hospital of Zhejiang Lishui, Lishui 323000, China; ^2^School of Pharmaceutical Sciences, Wenzhou Medical University, Wenzhou 325035, China

## Abstract

The systemic investigation of the metabolic pathways associated with the hepatic tumorigenesis is important to discover novel biomarkers and identify the potential pathogenesis. Here, the ^1^H nuclear magnetic resonance- (^1^H NMR-) based metabolomic analysis was used to monitor the whole process of rat diethylnitrosamine-induced HCC. Intraperitoneal administration of diethylnitrosamine (DEN) was used to induce primary HCCs in male Sprague-Dawley rats. Magnetic resonance imaging (MRI) examinations were performed to follow the tumor formation and growth in the liver and H&E staining was used to confirm MR imaging findings. The rats with DEN treatment and control rats without DEN were euthanized at the time points of 3, 8, and 15 weeks after the start of modeling. ^1^H NMR-based metabolomic analysis was used to explore hepatic metabolite changes and certify key metabolic pathways in the process of tumor tumorigenesis. Our MRI results depicted the formation of HCC nodules in ten rats 14 weeks after DEN injection which were confirmed by histology. Twenty-four different metabolites were identified and quantified by ^1^H NMR spectroscopy; OPLS-DA models and corresponding VIP plots analysis further identified ten metabolites associated with the abnormal metabolism. The aberrant glucose, lipid, and glutathione-glutamine-glutamate metabolism could be detected involving in the process of hepatic tumorigenesis, which provides an important evidence for the in-depth study of subsequent molecular mechanisms, especially the glutathione-glutamine-glutamate metabolism.

## 1. Introduction

Hepatocellular carcinoma (HCC) is the most common type of primary malignancy with an increasing incidence, and it is the third-leading cause of cancer-related deaths around the world [1]. A majority of HCCs were diagnosed at the advanced stage and lost the chance of curative treatments, such as surgical resection or orthotopic liver translation and the five-year survival rate after surgical resection remains dismally lower than 6% [2]. It was urgent to explore the key step of HCC for improving the long-term overall survival for patients.

With the broad investigations into the underlying molecular mechanism associated with the development of HCC, a series of crucial risk factors and molecular pathways involved in the hepatocarcinogenesis have been identified. Calvisi et al. [3] found that the ubiquitous activation of Ras and Jak/Stat pathways is essential for human HCC development. Ren et al. [4] detected 63 different proteins in liver tissues of HCC patients via proteomics analysis and found that the remarkable upregulation of phosphoglycerate mutase 1 (PGAM1) was strongly associated with the poor differentiation and decreased overall survival rates. However, very few studies have investigated the dynamic change of metabolomic characteristics over the whole process of hepatocarcinogenesis.

Tumor metabolomics, developing along with tumor proteomics and genomics, has been widely used to explore the metabolic profiles of tumorigenesis and elucidate the key metabolic pathways in different tumors, such as lung cancer [5], pancreatic cancer [6], and colorectal cancer [7]. The molecular and metabolic mechanisms of HCC have been investigated by metabolomics as well. Wang et al. [8] used ^1^H NMR-based metabolomics to explore the metabolite profiles of HCC formation and metastasis and found that the metabolism of glycine and choline was changed during HCC invasion and metastasis. However, Wang et al. were concerned with the metabolomic changes after the formation of HCC and lung metastasis of liver cancer; they did not pay attention to the metabolic changes at different time points in the process of liver cancer formation, and the changes of metabolic pathways at different time points for hepatocarcinogenesis are still unclear.

In the present study, DEN was used to establish the HCC model of rat and simulate the pathogenic process of clinical HCC [9]; the metabolic profiles at 3rd, 8th, and 15th week after DEN injection, which were used to mimic the three different stages of formation of hepatitis, cirrhosis, and HCC, were explored via ^1^H NMR-based metabolomic technique [10]. The key metabolic pathways involved in the hepatocarcinogenesis were investigated, with the aim of characterizing the metabolic profile in the course of the hepatocarcinogenesis.

## 2. Materials and Methods

### 2.1. Study Design

To explore dynamic change of metabolomic characteristics of hepatocarcinogenesis, the concentrations of different hepatic metabolites at the stages of HCC formation in DEN-treated rats were confirmed by ^1^H NMR-based metabolomic approach.

### 2.2. Animals

Sixty male Sprague-Dawley rats with the body weight of 180-200 g were obtained from Shanghai Laboratory Animal Co. Ltd. (SLAC, Shanghai, China) and housed in the SPF Laboratory Animal Center of Wenzhou Medical University (Wenzhou, China) with a regulated temperature of 24±2°C, relative humidity of 50±10%, and a 12/12-hour light/dark cycle. All rats were allowed free activity and fed with standard rat chow and tap water ad libitum. All the procedures were conducted in rats in the compliance with the National Institutes of Health Guide for the Care and Use of Laboratory Animals, as well as the guidelines of the Animal Welfare Act of China. The protocol for the animal experiments was approved by the Institutional Animal Care and Use Committee of Wen Zhou Medical University.

### 2.3. Animal Groups

All rats were randomly divided into DEN-treated group (n=30) and control group (n=30), with 10 rats being sacrificed at each time point in the two groups. Two rats in the control rats died unexpectedly at the sixth week of modeling, and one rat in the DEN-treated group died at the seventh week probably due to the DEN toxicity. The rats in the DEN-treated group were euthanized at the time points of 3 (n=10), 8 (n=9), and 15 weeks (n=10) after intraperitoneal DEN injection, and the numbers in corresponding control groups were 10, 8, and 10. DEN was dissolved in 0.09% sodium chloride solution at a concentration of 15mg/ml and kept in light-proof container. The rats in the DEN group received intraperitoneal injection of freshly prepared DEN solution at a dose of 5 ml/kg once a week for twelve weeks, and the rats in the control group were treated with equivalent volumes of 0.09% sodium chloride solution for the same period.

### 2.4. Magnetic Resonance Imaging to Monitor the HCC Formation in Rat Livers

The rats in the DEN-treated group were monitored by MR imaging every two weeks till the end points of study. Briefly, MR imaging examinations of rat livers were performed with 3T MR imaging system (Ingenia, Philips Healthcare, Best, Netherlands) by using a commercially available wrist coil, and images were acquired using the following sequences: T1WI, T2WI, and contrast-enhanced MRI. T1WI parameters were as follows: FOV, 60×40 mm; TR, 294 ms; TE, 15 ms; slice thickness, 2.0 mm; number of slice, 10. T2WI parameters were as follows: FOV, 60×40 mm,TR, 3000 ms; TE, 80 ms; slice thickness, 2.0 mm; number of slice, 10. The contrast agent Gd-DOTA (Dotarem, Guerbet SA, France) was manually injected at the dose of 30 *μ*mol/rat through tail vein within 2s, and the contrast-enhanced MRI parameters were as follows: FOV, 60×40 mm; TR, 400 ms; TE, 9.6 ms; slice thickness, 2mm; number of slice, 10; ACQ voxel, 0.3×0.3 mm; reconstruction voxel size, 0.268 mm.

### 2.5. Liver Sample Preparation and Metabolite Extraction

At each time point, the rats in each subgroup were fasted overnight with free access to water. The animals were sacrificed using decapitation, and the rat livers were harvested, which were cut into two pieces, with one-piece snap-frozen in liquid nitrogen, and then stored in −80°C freezer, and the other one was fixed in 4% paraformaldehyde, embedded in paraffin for further hematoxylin and eosin (H&E) staining. The frozen liver tissues were prepared for metabolite analysis, and the sample processing had been reported in our previous studies [11]; the liver tissues were cut into small pieces with each piece weighted approximately 400 mg and then extracted using the methanol-chloroform-water extraction method. The extracted supernatant lyophilized for 24 hours, and the tissue sample was redissolved in 500 *μ*l of D_2_O with 0.4 mmol/L trimethylsilyl-propionic-2,2,3,3d4-acid (TSP) and centrifuged at 10,000 g for 10 min at 4°C, and the supernatant was collected in a 5 mm NMR tube for NMR spectroscopy analysis.

### 2.6. Analytical Methodologies for Metabolite Profiling

All ^1^H NMR spectra were acquired using a 600 MHz Bruker Avance 600 spectrometer (Bruker BioSpin, Gmbh, Rheinstetten, Germany) with a 5 mm TXI probe operating at 600 MHZ; presaturation ‘zgpr' pulse program was used, 90° flip angle, a spectral width of 12kHz and 32K data points. The acquisition time was 2.65s per scan, and an additional 8s relaxation delay was used to ensure full relaxation. The total number of scans was 128. The spectra were zero-filled to 64K and an exponential line-broadening function of 0.3Hz was applied to the free induction decay prior to Fourier transformation. All spectra were corrected manually for phase and baseline and referenced to the chemical shift of the methyl peak of lactate (CH3, 1.33 ppm) using Topspin (v2.1 pl4, Bruker Biospin, Germany).

### 2.7. Metabolomics Data Processing

Before starting multivariate statistical analysis, each NMR spectrum (-0.04-10 ppm) was divided into integrated regions of 0.01 ppm and 0.0015 ppm width using the MATLAB (R2012a, The MathWorks Inc., Natick, MA, USA) after phase and baseline correction. The *δ*5.17–4.70 regions were excluded to eliminate the effect of the residual peak from suppressed water resonance [12]. The remained spectral segments were then normalized to the total sum of the spectral intensity to compensate for variations in total sample volume. The normalized integral values were then subjected to further analysis.

To distinguish metabolic pattern between the control group and DEN-treated group and characterize the changes of metabolic profile during hepatocarcinogenesis, principal component analysis (PCA) model and orthogonal partial least-squares discriminant analysis (OPLS-DA) model were performed to identify the separation between the control and DEN groups at different time points using the SIMCA 13.0 software (Umetrics, Umeå, Sweden). A more intuitive score plot of the first two principal components (PC1 and PC2) was obtained, in which each point represents an individual sample. Additionally, the corresponding variable importance in the projection (VIP) plot was adopted to identify the metabolites which contribute to the separation of the samples in the score plots.

### 2.8. Statistics Analyses

Statistical analysis of the normalized integral values was carried out using SPSS 18.0 software (version 18.0, SPSS). The continuous variables were expressed as mean±SD (standard deviation). Independent samples *t*-test was used to compare the differences in various metabolites between the two groups at different time points. The Benjamini-Hochberg false discovery rate (FDR) control was implemented to correct for multiple comparisons, and the FDR q-value threshold for significant markers was set at 0.05.

## 3. Results

### 3.1. Changes in Body Weight and MR Imaging Characteristics of Rat Liver with Tumors

**Figure 1:**
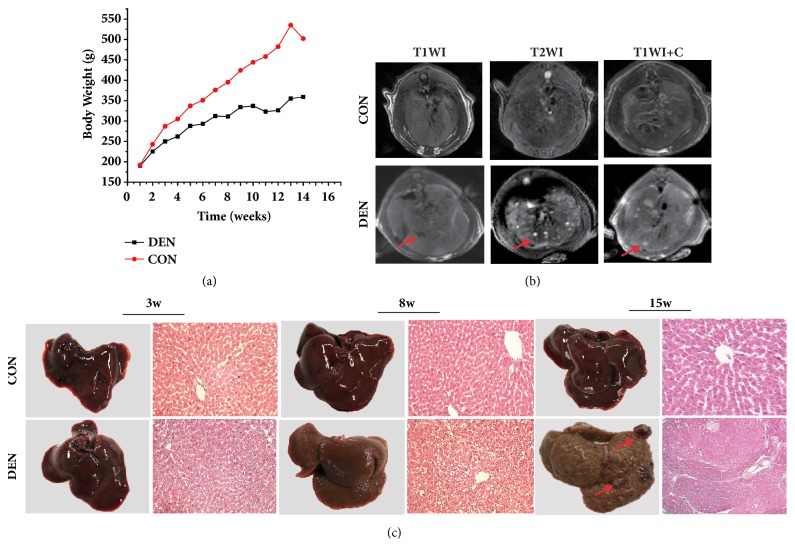
The development of primary HCCs in rats treated with intraperitoneal administration of DEN monitored by MR imaging. (a) The changes of body weight of the rats in the two groups at different time points. (b) The representative MR images of rat livers in two groups examined by MRI with different sequences (T1WI, T2WI, and dynamic contrast-enhanced T1WI) at 15th week after DEN injection. Both T2WI and DCE-T1WI detected a nodule in the liver with 15 weeks of treatment. (c) The histopathology of the livers treated with DEN shows the representative cirrhotic change of liver lobules at the 3rd week and 8^th^ week after DEN administration and the typical HCC nodules in the liver at the 15th week after DEN administration.

Figure 1(a) showed the changes of weight in the two groups at different time points. No significant difference of body weights was found between the two groups at the end of the first week since the intraperitoneal administration of DEN. The body weights of rats in the control group were increased over the observation time, but the rate in the DEN-treated group was slower than that of control group (13±4g versus 23.8±6g,* p*<0.05) and there was a significant difference in body weights between the two groups at the corresponding time points.

MR images did not detect any tumor in the rat livers with DEN inducement before the time point of 12 weeks after the initiation of intraperitoneal DEN administration. In the rat livers with 14 weeks of DEN treatment, the nodules in all the 10 rats were depicted, which manifests as 4-10mm low-intensity lesions (<1 cm) in the whole liver on T1WI images and presents as high-intensity signal on T2WI images and significant enhancement in contrast-enhanced T1WI images (Figure 1(b)). Histology also confirmed the MRI findings.

### 3.2. The Morphologic and Histologic Features of Rat Livers

The typical morphologic appearance of the livers in the two groups at 3, 8, and 15 weeks after DEN administration was shown in Figure 1(c). After three weeks of DEN treatment, we did not find any abnormalities in morphology. At the time point of 8 weeks after the DEN induction, the liver texture appeared to be stiff and inhomogeneous in color with diffuse nodules, which indicated the absence of liver cirrhosis. At the time point of 15 weeks, nodules were visualized in the parenchyma of livers, which were corresponding to the MRI findings and indicated the successful creation of HCC model of rats by DEN inducement.

The typical histologic appearances of livers by H&E staining were presented in Figure 1(c). The DEN-treated liver at the time point of 3 weeks showed the properties of early cirrhosis, manifesting as the enlarged hepatocytes with glycogen and fat deposition, but the structures of hepatic lobules remain intact. With the development of liver cirrhosis, increased numbers of necrotic hepatocytes were found in DEN-treated liver at the time point of 8 weeks and the formation of fibrous connective tissue also could be observed, which led to the structural disorganization of liver tissue. 15 weeks after DEN injection, a few tumor nodules were found in the DEN liver, shown as the infiltration of cancer cells in the liver parenchyma.

### 3.3. ^1^*H* NMR Spectral Analysis

**Figure 2:**
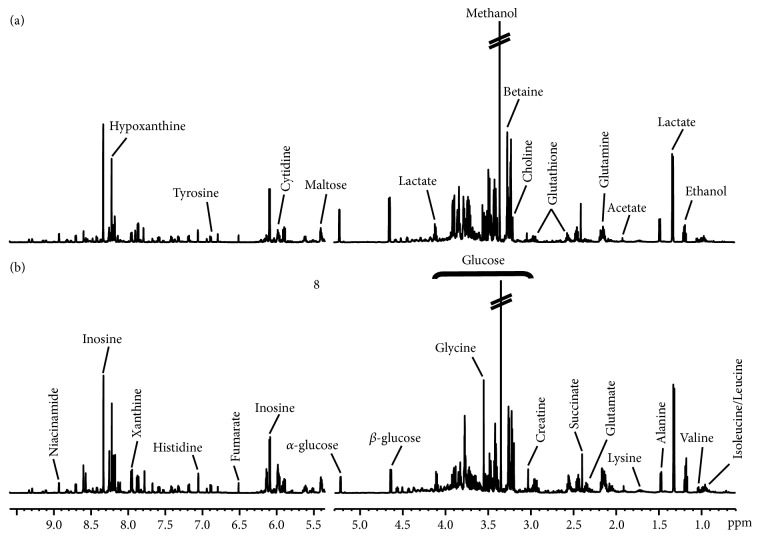
Representative spectra of the liver extracts produced by 600 MHz 1D ^1^H NMR in the control (a) and DEN-treated groups (b).

The representative ^1^H NMR spectra of liver samples obtained from control and DEN-treated rats were shown in Figure 2. The metabolite resonances signals were identified according to our previous study [13] and the 600 MHz library of the Chenomx NMR suite 7.0 (Chenomx Inc., Edmonton, Canada). The ^1^H NMR spectra of rat liver extracts enabled the simultaneous measurements of many endogenous metabolites. Twenty-four different metabolites were detected and quantified in our current study (Table 1), which were valine (*δ* 1.04), isoleucine (*δ* 0.94), leucine (*δ* 0.98), ethanol (*δ* 1.20), lactate (*δ* 1.33), alanine (*δ* 1.48), lysine (*δ* 1.70), acetate (*δ* 1.90), glutamate (*δ* 2.35), succinate (*δ* 2.40), glutamine (*δ* 2.45), creatine (*δ* 3.02), choline (*δ* 3.20), betaine (*δ* 3.30), glycine (*δ* 3.55), *α*-glucose (*δ* 5.22), *β*-glucose (*δ* 4.65), cytidine (*δ* 5.90), fumarate (*δ* 6.50), histidine (*δ* 7.10), hypoxanthine (*δ* 8.20), niacinamide (*δ* 7.60), glutathione (*δ* 2.90), and inosine (*δ* 8.30).

### 3.4. Characteristics of the Metabolic Changes

**Figure 3:**
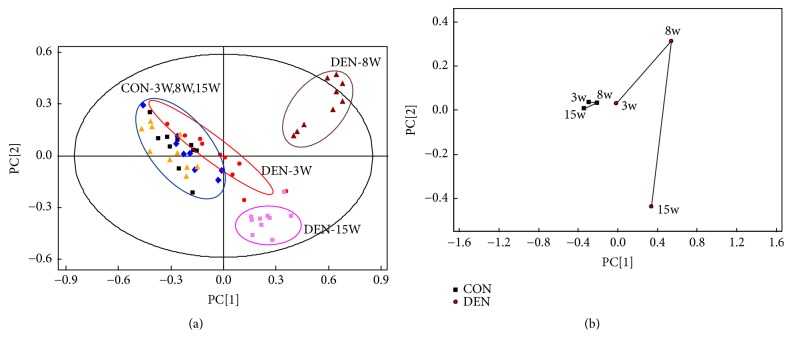
The metabolic profiles of the rat livers analyzed by PCA score plot at the different time points. (a) PCA score plot based on ^1^H NMR spectra of rat liver tissue samples in the DEN-treated group 3 weeks (red circle), 8 weeks (maroon triangle), and 15 weeks (pink square) after DEN treatment and the PCA score plot of liver tissue samples in the control group harvested at the corresponding time points of 3 weeks (black sqaure), 8 weeks (blue diamond) and 15 weeks (yellow triangle), showing that the control groups were gathered together, but the DEN-treated groups were separated from each other, indicating that the metabolic pattern of liver was significantly affected during hepatocarcinogenesis. (b) The liver PCA trajectory plot based on the mean ^1^H NMR spectra of liver tissue samples of the two groups, showing the influence of hepatocarcinogenesis on the metabolic profiles was stronger than that of the growth of the rats themselves.

The PCA scores plot (Figure 3(a)) and its corresponding mean trajectory (Figure 3(b)) were further applied to characterize the metabolic changes at the time points of 3, 8, and 15 weeks, based on ^1^H NMR spectra of the livers treated by DEN and the corresponding control groups. The analysis exhibited a distinct separation of the metabolites between DEN group and control group at the 8 and 15 weeks. In the first 3 weeks after the initiation of DEN treatment, the metabolites in the control rats converged in the “metabolic space”, which was shared with the metabolites of the corresponding DEN treatment rats. However, in the DEN groups, a distinctive change during the period from the 3 to 15 weeks was observed, which might be attributed to the process of hepatocarcinogenesis in the liver induced by DEN.

**Figure 4:**
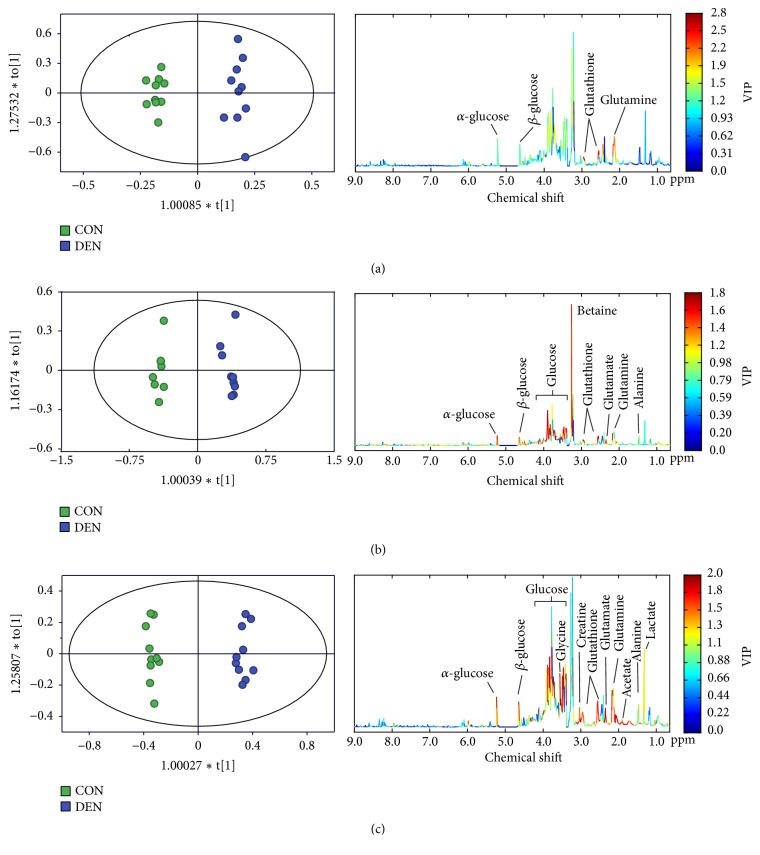
The OPLS-DA plots and VIP plots obtained from NMR-based hepatic metabolome in the control (green square) and DEN (purple square) groups at the time point of 3 weeks (a), 8 weeks (b), and 15 weeks (c), showing that the energy metabolism (glucose, lactate, and creatine), lipid metabolism (acetate), and amino acid metabolism (alanine, glycine, glutathione, glutamate, and glutamine) were contributed to the changes of metabolic pattern.

To further characterize the changes of the metabolic profiles in the process of hepatocarcinogenesis at different evolution stages, OPLS-DA model was used to examine the changes of metabolic pattern between the two groups at 3, 8, and 15 weeks after DEN injection and to identify the specific metabolites, which may contribute to the metabolic pattern change. OPLS-DA could clearly distinguish between the two groups regarding the metabolic profiles based on the liver metabolome at the three different stages (Figure 4). According to the corresponding VIP plots (Figure 4), a series of metabolites were identified, from which the metabolites (glucose, lactate, creatine, acetate, alanine, glycine, glutathione, glutamate, and glutamine) with a VIP value greater than 1.0 were selected for further analysis. Therefore, it implies that a series of abnormal metabolic mechanism may involve the process of hepatocarcinogenesis, including energy metabolism, lipid metabolism, and amino acid metabolism.

### 3.5. The Dynamic Changes of Metabolite Concentrations

**Figure 5:**
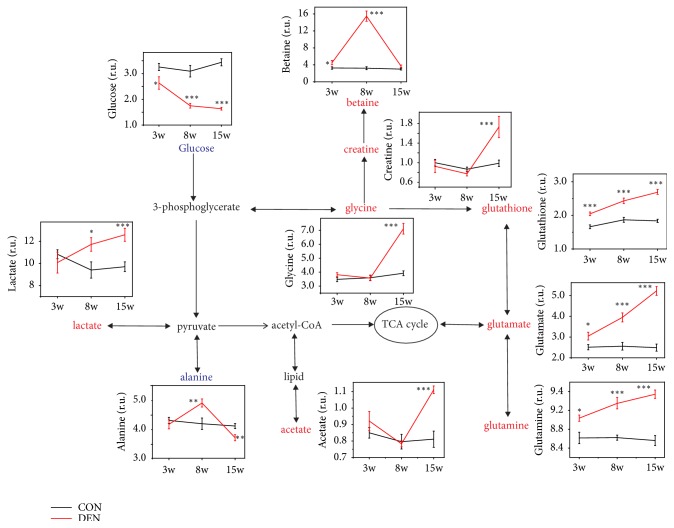
The metabolic pathways during hepatocarcinogenesis. The pathways based on the KEGG database and HMDB database show the interrelationships of the identified metabolic pathways of the rat liver with or without DEN treatment and the dynamic changes of the metabolites were illustrated in the graphs.

All the metabolites were further quantified (Table 1); 5 metabolites at 3 weeks, 15 metabolites at 8 weeks, and 13 metabolites at 15 weeks significantly remained after correction for multiple compassion (FDR q <0.05, shown in Table 1). These metabolites were further selected by VIP plots and analyzed the involved metabolic pathways.  Figure 5 showed the metabolic changes and their relevant pathways between two groups at the different time points. We found that the levels of glucose in DEN groups decreased over time, and the levels at different time points were all significantly lower than that in control groups, and the level of lactate in the liver with tumors increased over the weeks from 8 to15 weeks. Creatine is essential for the storage and transmission of phosphate-bond energy [14]; we found the significantly increased level of creatine in the livers of DEN group at 15 weeks, but there was no difference of creatine between the two groups at 3 weeks and 8 weeks. No content difference of lipid-related metabolites between two groups was identified, such as acetate. The level of betaine, an important methylation metabolite in the liver, significantly increased in DEN group at 8 weeks, but restored to the normal level at 15 weeks.

The content of amino acid metabolites, including alanine, glycine, glutathione, glutamate, and glutamine, between the two groups was significantly different as well. The level of alanine in DEN group was fluctuating over time, which was higher in the DEN-treated group than the control group at the time point of 8 weeks, but significantly lower than that in the control group at the time point of 15 weeks. It was interesting that the levels of glutathione, glutamate, and glutamine were increased significantly over time, and higher than that in the control group at different time points.

## 4. Discussion

Hepatocarcinogenesis has been characterized by an obvious multistep process associated with the accumulation of a series of genetic, epigenetic, and metabolic alterations during the initiation, promotion, and progression of cancer entities [15]. However, little was known about metabolic profile that involves hepatocarcinogenesis, although Wang et al. compared the difference in metabolic profile between HCC and normal tissues using the same method [8]. In the present study, ^1^H NMR-based metabolomics were used to characterize the metabolic changes involved in hepatocarcinogenesis, focusing on the stages of early liver damage, liver cirrhosis, and liver cancer and identifying the key metabolic pathways during the process.

### 4.1. DEN-Induced Hepatocellular Carcinomas in Rats

MRI examination detected the hepatocellular nodules in 10 rat livers 14 weeks after the initiation of DEN induction, which was confirmed by histopathology. At 3 weeks, mild pathological changes could be observed in the liver, manifesting as inflammation, cellular damage, and steatosis, but no nodules with abnormal signals were found in MR images. In the later period at 8th week after DEN induction, histology by H&E staining reveals the structural alteration of hepatic parenchyma characterized by the distortion of hepatic lobules with regenerative hepatocytes and fibrosis, but neither MRI nor histopathology detected obvious tumor nodules. At the time point of 15 weeks after the DEN treatment, more apparent structure alterations and evidence of cirrhosis were observed in the liver accompanied by the formation of tumor nodules with abnormal MRI signals confirmed by histology as HCC nodules. Our rat HCC model demonstrated similar MR imaging and histologic appearance described in a previous study [16].

### 4.2. Energy Metabolism during Hepatocarcinogenesis

Sufficient energy supply is vital for cancer cell growth and proliferation, and it has been confirmed that rapidly growing tumors would adapt the energy demands during the process tumorigenesis by inducing adequate blood supply [17]. Glucose is the predominant nutrient source, which can provide the energy support through energy metabolism of either tricarboxylic acid cycle (TCA) or glycolysis. Increased glycolysis has been regarded as one of the earliest hallmarks of cancer development [18]. Our study found that the levels of glucose in DEN group were significantly decreased over time, as compared to the control group. There is an enlarging difference of glucose level in the livers between the two groups, indicating the increasing energy demand during the process of tumorigenesis in the rat group with DEN treatments, which is different from the energy supply in the normal cells that most of the glucose enter into the tricarboxylic acid (TCA) cycle via the pathway of glycolysis. However, due to the Warburg effect [19, 20], energy supply may be provided by the pathway of glycolysis which yields great amount of lactate or alanine via pyruvate during tumorigenic transformation. Our study found that the level of lactate in DEN group was significantly elevated at the time point of 8 weeks and 15 weeks, as compared to the control group, but there was no difference of lactate level at the early stage of DEN induction. Wang et al. also found that the glucose level in HCC tissues was lower than that in normal liver tissues, but the lactate was higher [8].

Creatine is another metabolite participating in energy production, which mainly accumulate in the liver and can help to maintain the fluctuating energy demands [21]. In this study, significantly elevated content of creatine was detected in the DEN-treated liver at the 15^th^ weeks, but no difference was found at the 3^rd^ and 8^th^ week as compared to the level in the normal livers, suggesting that the increased level of creatine was associated with the malignant transformation of hepatocytes as that happening in HCC tissue.

### 4.3. Lipid Metabolism during Hepatocarcinogenesis

Liver is the pivotal organ harboring the metabolism of lipogenesis. The aberrant metabolism of lipid has been reported to be associated with many pathological processes, such as hepatic steatosis and malignant transformation [22]. Muir et al. [23] showed that the long-chain N^6^-polyunsaturated fatty acids may play an important role in the pathogenesis of nonalcoholic steatohepatitis (NASH) and in the process of tumorigenesis. The dynamic change of acetate, the end product of lipid metabolism, could indirectly reflect the activity of hepatic lipid metabolism. The significant increase of acetate content in the liver was observed in the rat livers of DEN-treated group at the 15^th^ week, indicating that the metabolism of lipid involves the sequential stages of HCC development by providing the supplemental energy source for the proliferation of HCC tumor cells. This result was consistent with Wang et al. who observed an increase in acetate in HCC tissues [8]. Interestingly, no changes in acetate levels were observed at 3 and 8 weeks, further suggesting that abnormalities in lipid metabolism were often accompanied by the progression of liver cancer cells and acetate could be used as a potential biomarker for early HCC.

### 4.4. Hepatic Glutathione-Glutamate-Glutamine Metabolism during Hepatocarcinogenesis

Glutathione, working as the main nonprotein thiol in mammalian cells, participates in multiple physiologic process, including antioxidant defense and cell growth [24]. The level of glutathione was confirmed to be associated with the proliferation of human HCC cell line in vitro and the increased level of glutathione was found in human HCCs, as a result of upregulated expression of *γ*-glutamylcysteine synthetase heavy subunit (GCS-HS) and glutathione synthetase (GS) [25]. Our study showed similar phenomenon that the level of glutathione increased over time during the observed process of hepatocarcinogenesis and was significantly higher than that in the control group at different stages, indicating that increased GSH level could facilitate the growth of liver cancer cells.

Glutamate, a nonessential amino acid, provided the carbon source to support cell proliferation [26] and played an important role in hepatic tumorigenesis. Prickett et al. [27] reported that glutamate could stimulate the tumor growth and proliferation by activating the mitogen-activated protein kinase and phosphoinositide 3-kinase/Akt pathways. Furthermore, the production of glutamate and/or the activity of glutamate receptor could affect the invasion of breast, laryngeal, and pancreatic cancers [28]. The abnormal changes of glutamate could be observed in alcoholic cirrhosis, HCCs, and multiple steps of hepatocarcinogenesis [29]. In our study, the level of glutamate was substantially increased over the HCC tumorigenesis, which may be attributed to increasingly transformed glutathione for meeting the needs of carbon supply by fast proliferating tumor cells.

Glutamine, another nonessential amino acid, can be catalyzed to form glutamate by glutaminolysis, which plays a central role in the metabolism of tumor cells [30] and can augment the cancer progression by regulating the activities of multiple oncogenes and tumor suppressing genes [31]. Osada et al. [32] showed that the expression of glutamine synthetase may have a close relationship with the metastatic potential in HCC and was also the potential biomarker for high-risk HCC, as similarly demonstrated in another study [33]. Our study showed the significant higher glutamine in the rat liver with DEN treatment at different stages of tumorigeneses than that in the corresponding control groups, suggesting that the activity of glutamine synthetase involves the process of hepatocarcinogenesis. Wang et al. also observed that glutamine level was elevated in HCC tissues compared to controls.

The three metabolites of glutathione, glutamate, and glutamine were in the same metabolic pathway, and the levels increased significantly with the course of hepatocarcinogenesis, which gave us the preliminary conclusion that the glutathione-glutamate-glutamine metabolism may play an important role in the process of hepatocarcinogenesis.

### 4.5. Other Metabolites

Glycine, a nonessential amino acid, having the potential of antiangiogenic effects, could be synthesized in liver or kidneys from a glycolytic intermediate [34], 3-phosphoglycerate. A prior study demonstrated that glycine has the potential of antitumor effect. The study by Amin et al. [35] showed that dietary glycine could exert some antitumor effect of retarding 15% tumor growth and a potent antiangiogenic effect of decreasing 20% of tumor microvessel density in the breast adenocarcinoma of a rat model. Our study observed the increased level of glycine in the livers of DEN-treated rats at the period of HCC formation, which also may be closely related to the self-protection of the liver. Wang et al. [8] also detected an increase level of glycine in early HCC.

Betaine, one of the important carbon sources for the conversion of different metabolites, has been confirmed to participate in the biosynthesis of nucleotides and DNA methylation, which may play a crucial role in the process of carcinogenesis [36]. The previous study [37] found that betaine could protect liver from fibrogenesis by attenuating the hepatotoxicity and fibrosis induced by dimethylnitrosamine. Our study found that the level of betaine significantly increased at 3^rd^ week and 8^th^ week during the period of DEN treatment, which could be explained by the underling self-protection mechanism of cells by producing high level betaine to counteract the cellular toxicity of DEN. Alanine was the major gluconeogenic precursor and fluctuated in DEN group during tumorigenesis, and this was inconsistent with Wang et al.'s observation that alanine levels increase in liver tumor tissue [8]. We believe that the results in the present study were related to the energy demand of different stages of hepatocarcinogenesis, and the level of alanine was also closely related to the stage of tumor development, which may result in some differences between the two studies.

Our study has limitations. First, we initially characterized the dynamic changes of the key metabolites associated with the multistep hepatic tumorigenesis, but the pivotal metabolic pathway, such as the glutathione-glutamate-glutamine metabolism, which may involve the tumorigenesis needs to be further elucidated in the future study. Secondly, multianalytical techniques with the advantages of being able to detect more metabolites need to be developed. We will analyze human cirrhotic liver and HCC samples incised from the patients with HCC to characterize the metabolic profiles of hepatic tumorigenesis.

## 5. Conclusion

In summary, we initially profiled the aberrant energy, lipid, and glutathione-glutamate-glutamine metabolisms over the course of rat hepatic cancer development induced by DEN. The biomolecules in the metabolic pathway which govern the glutathione-glutamate-glutamine metabolism in the early stage of hepatic tumorigenesis may be the potential early biomarker for identifying HCC. This study made us have a deeper understanding of the changes in metabolic pathways during the formation of liver cancer.

## Figures and Tables

**Table 1:** Comparison of different metabolite levels between the control and DEN group during the different time period after DEN treatment.

Metabolites	Chemical shift	3-Weeks		8-Weeks		15-Weeks	
		CON	DEN	CON	DEN	CON	DEN
Energy metabolism							
*α*-Glucose	*δ* 5.22	3.26±0.14	2.64±0.24*∗*	3.09±0.23	1.75±0.09*∗∗∗*	3.44±0.14	1.64±0.05*∗∗∗*
*β*-Glucose	*δ* 4.65	3.4±0.17	2.77±0.26	3.21±0.27	1.85±0.10*∗∗∗*	3.54±0.22	1.66±0.05*∗∗∗*
Succinate	*δ* 2.40	7.91±0.46	8.03±0.43	7.27±0.37	8.36±0.37	6.55±0.42	6.50±0.30
Fumarate	*δ* 6.50	0.22±0.03	0.27±0.02	0.26±0.02	0.29±0.02	0.24±0.02	0.25±0.03
Lactate	*δ* 1.33	10.88±0.41	10.08±0.95	9.40±0.73	11.72±0.62*∗*	9.70±0.44	12.60±0.59*∗∗∗*
Creatine	*δ* 3.02	1±0.07	0.92±0.13	0.87±0.04	0.77±0.04	0.99±0.07	1.73±0.22*∗∗∗*
Lipid metabolism							
Acetate	*δ* 1.90	0.85±0.03	0.92±0.06	0.80±0.04	0.78±0.02	0.81±0.05	1.11±0.02*∗∗∗*
Amino acid metabolism							
Valine	*δ* 1.04	0.88±0.03	0.94±0.05	0.86±0.04	0.84±0.03	0.83±0.05	1.09±0.04*∗∗∗*
Isoleucine	*δ* 0.94	0.56±0.02	0.6±0.04	0.58±0.03	0.42±0.02*∗∗*	0.54±0.03	0.57±0.02
Leucine	*δ* 0.98	1.27±0.03	1.41±0.08	1.26±0.04	1.04±0.04*∗∗*	1.32±0.08	1.47±0.04
Glutamate	*δ* 2.35	2.51±0.13	3.05±0.18*∗*	2.56±0.18	3.95±0.22*∗∗∗*	2.49±0.17	5.22±0.21*∗∗∗*
Alanine	*δ* 1.48	3.8±0.11	3.66±0.16	3.68±0.19	4.39±0.14*∗∗*	3.60±0.09	3.20±0.10*∗∗*
Lysine	*δ* 1.70	2.7±0.08	2.97±0.17	2.66±0.13	2.44±0.07	2.67±0.16	3.64±0.09*∗∗∗*
Betaine	*δ* 3.30	3.27±0.27	4.32±0.5	3.21±0.35	15.48±1.2*∗∗∗*	3.00±0.22	3.68±0.28
Histidine	*δ* 7.10	0.89±0.03	1.02±0.04*∗*	1.00±0.08	1.03±0.03	0.88±0.04	1.07±0.05*∗∗*
Glutamine	*δ* 2.45	8.61±0.12	9.03±0.07*∗*	8.62±0.05	9.34±0.11*∗∗∗*	8.56±0.11	9.54±0.09*∗∗∗*
Glycine	*δ* 3.55	3.48±0.16	3.82±0.14	3.60±0.19	3.57±0.19	3.92±0.17	7.11±0.38*∗∗∗*
Other metabolites							
Glutathione	*δ* 2.90	1.66±0.06	2.05±0.05*∗∗∗*	1.87±0.07	2.43±0.08*∗∗∗*	1.84±0.05	2.69±0.08*∗∗∗*
Ethanol	*δ* 1.20	3.44±0.36	3.78±0.39	3.31±0.40	3.29±0.34	3.40±0.27	3.75±0.39
Choline	*δ* 3.20	0.76±0.07	0.74±0.06	0.48±0.04	0.38±0.02*∗*	0.82±0.11	0.90±0.07
Cytidine	*δ* 5.90	0.24±0.01	0.23±0.01	0.24±0.01	0.18±0.01*∗∗*	0.16±0.01	0.15±0.01
Hypoxanthine	*δ* 8.20	2.52±0.08	2.62±0.13	2.63±0.10	1.82±0.06*∗∗∗*	2.45±0.13	2.18±0.14
Niacinamide	*δ* 7.60	0.47±0.06	0.44±0.04	0.41±0.02	0.31±0.03*∗*	0.63±0.06	0.59±0.04
Inosine	*δ* 8.30	0.57±0.02	0.59±0.02	0.59±0.03	0.44±0.02*∗∗∗*	0.59±0.03	0.53±0.02

Values are expressed as mean ± SE. CON, control group; DEN, DEN-induced group. Significant levels: *∗p*< 0.5, *∗∗p*< 0.01, and *∗∗∗p*< 0.001, compared with control group, which were FDR correction for p values calculated from independent samples *t*-test.

## Data Availability

The data used to support the findings of this study are available from the corresponding author upon request.
